# Molecular Genetics of Bartter Syndrome: Bridging Genotype–Phenotype Correlations and Precision Therapeutics

**DOI:** 10.3390/cimb48040422

**Published:** 2026-04-19

**Authors:** Lina Zhu, Yang Li, Yiyao Bao

**Affiliations:** Department of Surgical Intensive Care Unit, Children’s Hospital, Zhejiang University School of Medicine, National Clinical Research Center for Children and Adolescents’ Health and Diseases, Hangzhou 310052, China; 6513282@zju.edu.cn (L.Z.); 6523217@zju.edu.cn (Y.L.)

**Keywords:** Bartter syndrome, molecular genetics, genotype–phenotype correlation, renal tubular disorders, precision medicine, ion channels

## Abstract

Bartter syndrome (BS) represents a group of rare, autosomal recessive renal tubular disorders characterized by hypokalemic hypochloremic metabolic alkalosis, secondary hyperaldosteronism, and normal to low blood pressure. The underlying pathophysiology is primarily driven by defects in critical ion transport proteins or channels localized within the thick ascending limb of the loop of Henle, leading to impaired salt reabsorption. Recent advances in molecular genetics have refined the classification of Bartter syndrome. Current evidence supports SLC12A1, KCNJ1, CLCNKB, BSND, and MAGED2 as the core disease genes within the contemporary BS spectrum, with MAGED2 causing a distinct X-linked transient antenatal form. In contrast, gain-of-function CASR variants, historically labeled “type V Bartter syndrome”, are now more appropriately described as CaSR-associated Bartter-like phenotypes within the broader spectrum of disorders of calcium homeostasis. Despite significant progress, two primary research limitations remain. First, fully elucidating genotype–phenotype correlations and overcoming diagnostic complexities continues to be highly challenging due to substantial phenotypic overlap and genetic heterogeneity. Compounding these diagnostic hurdles is the equally critical challenge of understanding mutation-driven pathogenic mechanisms to develop viable clinical interventions. This review systematically summarizes the current molecular genetic landscape of BS to address these gaps. We highlight the relationships between specific genetic variants and clinical manifestations, delve into molecular pathophysiology including protein misfolding and trafficking defects, and explore emerging therapeutic approaches such as molecular chaperones. By integrating genetic and clinical data, this work aims to provide a comprehensive framework to facilitate precise diagnosis and individualized treatment strategies, ultimately advancing precision medicine in the management of Bartter syndrome.

## 1. Introduction

Bartter syndrome (BS) represents a group of rare inherited salt-losing tubulopathies characterized by hypokalemic metabolic alkalosis, hyperreninemia, and hyperaldosteronism, with clinical manifestations ranging from severe antenatal onset to milder adult presentations [1,2]. This phenotypic heterogeneity poses significant challenges for clinical diagnosis and management [3]. At the molecular level, BS is genetically heterogeneous, with mutations identified in several genes encoding key renal tubular transport proteins and their regulatory subunits [4,5,6]. These causative genes include SLC12A1 encoding the Na^+^-K^+^-2Cl^−^ cotransporter NKCC2 (type I BS), KCNJ1 encoding the renal outer medullary potassium channel ROMK (type II BS), CLCNKB encoding the basolateral chloride channel ClC-Kb (type III BS), and BSND encoding barttin, an accessory subunit essential for ClC-K channel function (type IV BS). In addition, MAGED2 is now recognized as causing a distinct X-linked transient antenatal Bartter syndrome [7,8]. By contrast, gain-of-function CASR variants are better regarded as CaSR-associated Bartter-like phenotypes rather than a stable canonical BS subtype [2].

Recent advances in high-throughput sequencing technologies, particularly targeted gene panel testing for renal tubular disorders, have substantially improved the diagnostic yield for BS and related tubulopathies [9,10]. This has led to the identification of numerous novel pathogenic or likely pathogenic variants, expanding the mutational spectrum and refining the molecular classification of the disease. However, relying solely on advancing sequencing technologies is insufficient to fully overcome the barriers of clinical classification [11]. Compounding the diagnostic challenges of phenotypic overlap is an equally critical limitation: a distinct gap in our understanding of how these specific genetic mutations drive cellular dysfunction. Without elucidating the pathophysiological basis of the disease and the underlying genotype–phenotype correlations, the development of viable clinical interventions remains severely restricted [12]. This updated classification has important clinical implications. MAGED2-related disease typically presents with severe fetal polyhydramnios, prematurity, and marked antenatal salt wasting, followed by partial or complete postnatal resolution in many survivors [7,8]. In contrast, CaSR-related disease overlaps substantially with autosomal dominant hypocalcemia and other disorders of calcium homeostasis, making its placement within the traditional Bartter subtype numbering system increasingly unsatisfactory.

To address this mechanistic gap, recent studies focusing on type I BS have shown that SLC12A1 variants impair NKCC2 through several distinct molecular routes, including defective folding, ER retention, enhanced ER-associated degradation (ERAD), impaired forward trafficking, and reduced apical membrane delivery [13,14]. Functional studies indicate that some missense variants are retained in the endoplasmic reticulum, where they fail to complete normal maturation and become susceptible to quality-control surveillance and proteasomal degradation [13]. However, not all NKCC2 defects are explained solely by ER retention. Certain variants appear to permit partial maturation yet still show deficient surface expression, suggesting that post-ER and possibly post-Golgi trafficking steps may also contribute to the loss of transporter activity.

Complementary molecular studies have identified specific chaperones that differentially regulate NKCC2 biogenesis and stability, underscoring the importance of proteostasis in type I BS [15]. For example, the stress-inducible heat shock protein 70 (Hsp70) and the chaperone stress 70 protein (STCH) exert opposing effects on NKCC2 maturation. Hsp70 promotes folding and stability, whereas STCH facilitates degradation through proteasomal and lysosomal pathways [15]. These findings suggest that the cellular fate of mutant NKCC2 is determined not only by the mutation itself but also by the balance between folding assistance, ER quality control, and downstream trafficking competence.

These mechanistic insights have also stimulated interest in targeted rescue strategies, particularly for trafficking-defective NKCC2 variants [13]. Experimental studies suggest that chemical chaperones such as 4-phenylbutyric acid (4-PBA) may improve folding, ER exit, and membrane delivery of selected SLC12A1 mutants in vitro. This observation is mechanistically informative because it supports the concept that at least a subset of type I BS variants act through correctable biosynthetic defects rather than through complete absence of protein production.

Beyond NKCC2-related BS, mechanistic heterogeneity is also evident in other BS genes. Mutations in CLCNKB and BSND impair basolateral chloride transport through variable effects on channel expression, membrane localization, gating, and interaction with barttin [5,16]. Likewise, KCNJ1-related type II BS should not be viewed solely as a channelopathy defined by altered potassium conductance. Experimental work indicates that some ROMK mutants undergo misfolding, ER retention, and accelerated degradation, whereas others reach the membrane but display impaired gating or reduced channel activity [5,17]. This distinction is important because it suggests that type II BS, like type I BS, may arise from mechanistically distinct subclasses of variants rather than from a single uniform molecular defect.

Collectively, molecular genetic research has substantially advanced our understanding of BS pathogenesis and highlighted the need to integrate genetic diagnosis with functional interpretation [4,18,19]. In this review, we therefore focus on the molecular basis of disease, the limits of current genotype–phenotype correlations, and the translational prospects of mechanism-informed therapeutic strategies [20,21,22].

## 2. Molecular Genetics and Pathophysiology

### 2.1. Causative Genes and Renal Ion Transport

**Figure 1:**
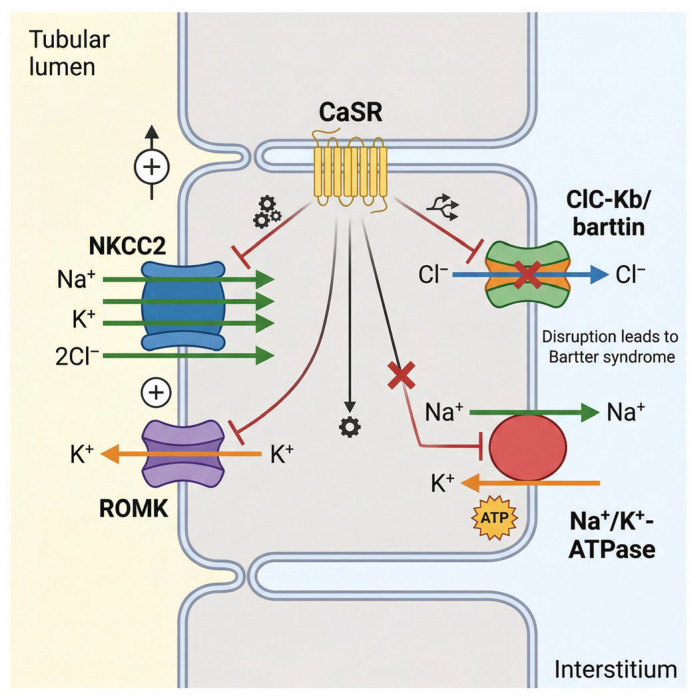
Molecular physiology and ion transport mechanisms in the thick ascending limb (TAL) of the loop of Henle. NKCC2, located at the apical membrane, mediates Na^+^-K^+^-2Cl^−^ cotransport from the tubular lumen into TAL epithelial cells. ROMK recycles K^+^ back into the lumen to sustain NKCC2 activity and contributes to the lumen-positive transepithelial voltage. On the basolateral side, ClC-Kb, together with its essential accessory subunit barttin, mediates chloride efflux into the interstitium, while the Na^+^/K^+^-ATPase maintains the electrochemical gradients required for ongoing salt reabsorption. The calcium-sensing receptor (CaSR) modulates ion transport and calcium handling in this segment. Disruption of these transport pathways underlies the major molecular forms of Bartter syndrome and related Bartter-like phenotypes.

Bartter syndrome (BS) constitutes a genetically heterogeneous group of salt-losing renal tubulopathies predominantly caused by defects in electrolyte transport within the thick ascending limb (TAL) of the loop of Henle and closely related nephron segments. This physiological disruption leads to the cardinal manifestations of hypokalemic metabolic alkalosis, secondary hyperaldosteronism, and normal-to-low blood pressure [23]. The contemporary molecular framework includes pathogenic variants in SLC12A1, KCNJ1, CLCNKB, BSND, and MAGED2 [7,8]. These genes affect critical transport proteins or regulatory pathways involved in TAL function. By contrast, gain-of-function CASR variants may produce a Bartter-like phenotype, but because they primarily disturb calcium-sensing pathways and overlap with disorders of calcium homeostasis, they are more appropriately discussed as related noncanonical entities rather than a core BS subtype, as shown in Figure 1.

The physiological engine of the TAL is primarily driven by the Na^+^-K^+^-2Cl^−^ cotransporter (NKCC2), which is encoded by the SLC12A1 gene. NKCC2 is exclusively localized to the apical membrane of the TAL cells, where it orchestrates the active, electroneutral reabsorption of sodium, potassium, and chloride ions directly from the tubular lumen. This transepithelial transport process is absolutely essential for the generation and maintenance of the corticomedullary osmotic gradient, which in turn dictates the kidney’s urinary concentrating capacity [24]. Loss-of-function mutations within the SLC12A1 gene profoundly abolish this transport activity, culminating in Bartter syndrome type I (BS I). Given the indispensable role of NKCC2 in fetal fluid balance and early renal salt handling, neonates afflicted with BS I typically present with severe antenatal manifestations [8,25,26]. These critical signs include massive polyhydramnios, fetal polyuria, and premature delivery, and affected infants often suffer from profound postnatal electrolyte disturbances and severe growth retardation.

The continuous and high-capacity function of NKCC2 is biochemically coupled to the activity of the renal outer medullary potassium channel (ROMK), encoded by the KCNJ1 gene. ROMK is prominently expressed on the apical membrane of both TAL cells and the principal cells of the collecting duct. Its fundamental physiological role is to facilitate the dynamic recycling of potassium ions back into the tubular lumen [27]. Because the luminal concentration of potassium is natively low, this recycling mechanism is an absolute prerequisite to sustain the continuous electrogenic activity of NKCC2 and to drive distal potassium secretion. Genetic mutations in KCNJ1 impair this essential recycling loop, resulting in Bartter syndrome type II (BS II). Clinically, BS II frequently manifests in the neonatal period with a paradoxical transient hyperkalemia, which eventually transitions into the hallmark hypokalemic metabolic alkalosis. Furthermore, BS II is strongly characterized by the early onset of nephrocalcinosis, a hallmark feature reflecting severely disturbed renal calcium handling secondary to the paralyzed sodium and chloride reabsorption pathways [28].

To maintain strict intracellular electroneutrality and complete the transepithelial salt reabsorption process, chloride ions must efficiently exit the TAL cells into the surrounding interstitium. This critical basolateral efflux is mediated by the voltage-gated chloride channel ClC-Kb, which is encoded by the CLCNKB gene and expressed predominantly on the basolateral membrane of TAL cells, as well as in the distal convoluted tubule [29]. Mutations localized within CLCNKB precipitate Bartter syndrome type III (BS III), frequently referred to as classic Bartter syndrome. Epidemiologically, BS III represents the most prevalent subtype within Asian populations and is clinically notorious for its substantial phenotypic variability. Patients may exhibit a spectrum of severity ranging from mild, late-onset electrolyte imbalances to severe, early-onset growth retardation and hypokalemia. This extreme clinical heterogeneity is largely dictated by the diverse repertoire of mutation types, ranging from missense and nonsense variants to large whole-gene deletions, that variably impact the channel’s function and basolateral expression [30,31], as shown in Table 1.

The functional integrity of the ClC-Kb channel relies heavily on barttin, an essential accessory β-subunit encoded by the BSND gene. Barttin is strictly required for the proper intracellular trafficking, stable basolateral membrane localization, and optimal gating function of both ClC-Ka and ClC-Kb chloride channels. Pathogenic mutations in BSND cause Bartter syndrome type IV (BS IV). Because barttin is co-expressed in both the renal tubules and the inner ear, BS IV presents with a unique, devastating dual phenotype: classical severe renal salt wasting combined with congenital sensorineural deafness. This striking extrarenal manifestation underscores the indispensable role of barttin in systemic chloride channel homeostasis across different organ systems [5,32].

Finally, the calcium-sensing receptor (CaSR), a G-protein coupled receptor encoded by the CASR gene, adds a vital layer of regulatory complexity to TAL physiology. CaSR tightly regulates parathyroid hormone secretion and directly modulates renal calcium and salt handling. Gain-of-function CASR mutations can produce a Bartter-like phenotype characterized by hypokalemic metabolic alkalosis together with hypocalcemia and hypercalciuria. However, because this condition overlaps substantially with autosomal dominant hypocalcemia and related disorders of calcium homeostasis, it is now more appropriately regarded as a CaSR-associated Bartter-like phenotype rather than a stable canonical BS subtype [33].

### 2.2. Molecular Pathogenic Mechanisms

The genetic mutations underlying Bartter syndrome (BS) are highly diverse, encompassing a wide array of genetic lesions including missense, nonsense, frameshift, and splice-site mutations, as well as large gene deletions [34]. The specific mutation spectrum varies significantly among different BS subtypes and distinct ethnic populations. For instance, whole-gene deletions of the CLCNKB gene represent the most common mutations identified in Chinese patients presenting with BS type III, underscoring the critical importance of structural variants in the disease’s etiology [35,36]. The overall complexity of genotype–phenotype correlations is further compounded by the frequent presence of compound heterozygous mutations, large genomic deletions, and complex structural rearrangements. Advanced long-read sequencing technologies have successfully uncovered multiple distinct deletion alleles within CLCNKB, including complex rearrangements and hybrid genes formed with CLCNKA, which significantly contribute to phenotypic variability and overall disease severity. Notably, a common transposition haplotype involving the 3′-untranslated region (3′-UTR) of CLCNKB has been shown to predispose individuals to recurrent genomic deletions, highlighting the profound role of local genomic architecture in mutation susceptibility [37].

At the molecular and cellular levels, the pathogenic mechanisms that disrupt renal ion transport in Bartter syndrome can be broadly categorized into three overlapping functional classes: protein expression defects, membrane trafficking or localization abnormalities, and direct loss of ion transport activity [38]. However, this framework should be interpreted cautiously. Many variants do not fit neatly into a single category, and the strength of evidence differs substantially across genes and mutations. In several instances, mechanistic conclusions are derived primarily from heterologous expression systems or single-variant functional studies rather than from patient-derived renal tissue, which limits the certainty with which these mechanisms can be generalized.

#### 2.2.1. Protein Expression Defects and the ERAD Pathway

Protein expression defects arise when pathogenic variants reduce the effective abundance of a transporter or channel through impaired biosynthesis, abnormal folding, or accelerated degradation. Among the best-studied examples are selected missense variants in SLC12A1 and KCNJ1, which can destabilize NKCC2 or ROMK during endoplasmic reticulum (ER) processing and promote recognition by ER quality-control pathways [5,17]. Nevertheless, the available evidence is not uniform across variants. Most ERAD-related conclusions are based on a limited number of experimentally characterized mutations, and these findings should not be extrapolated to all missense changes within the same gene.

Misfolded proteins may be retained within the ER and targeted for degradation through the ER-associated degradation (ERAD) pathway, thereby reducing mature transporter abundance at the cell surface. This model is biologically plausible and supported by functional studies, but several caveats deserve emphasis. First, many studies rely on overexpression systems in nonrenal cell lines, which may not fully reproduce the proteostatic environment of TAL cells. Second, different mutations within the same gene may produce distinct molecular consequences, ranging from severe ER retention to partial maturation with residual activity. Third, direct links between a specific ERAD phenotype and long-term clinical severity remain incompletely defined. Therefore, ERAD should be viewed as an important but mutation-selective mechanism rather than a universal explanation for all BS-associated protein defects [13,14].

#### 2.2.2. Membrane Localization and Trafficking Abnormalities

Membrane localization defects constitute a related but distinct pathogenic mechanism in which mutant proteins are synthesized but fail to reach the appropriate apical or basolateral membrane domain. Certain CLCNKB variants exemplify this pattern, showing impaired delivery of ClC-Kb to the basolateral membrane despite detectable protein expression [39]. Importantly, however, the trafficking literature remains uneven. For some variants, the available data support defective ER exit, whereas for others the abnormality may involve later post-Golgi processing, membrane stability, or abnormal interaction with partner proteins such as barttin. In addition, the distinction between “expression defects” and “trafficking defects” is often blurred experimentally, because reduced surface abundance may result from multiple sequential abnormalities. A more cautious interpretation is therefore warranted when assigning a single trafficking mechanism to a variant without comprehensive biochemical and localization analyses.

A further mechanism contributing to defective protein localization and loss of function is aberrant pre-mRNA splicing. Variants affecting canonical splice sites, nearby intronic regions, or even exonic residues can induce exon skipping, intron retention, or cryptic splice-site activation in genes such as CLCNKB and SLC12A1 [40]. These findings are highly relevant, but they should also be interpreted in light of methodological limitations. Much of the current evidence derives from minigene assays or indirect transcript analyses rather than from native renal tissue, and the extent to which abnormal transcripts are expressed in vivo may vary. In addition, splice-disrupting variants do not all produce complete loss of function; some may allow low-level normal transcripts or partial residual activity. Accordingly, splicing abnormalities should be discussed as a spectrum of molecular defects rather than a single uniform mechanism.

#### 2.2.3. Functional Loss of Ion Transport Activity

The third major category comprises variants that primarily impair intrinsic transport or channel-gating function without causing a major defect in total protein abundance or membrane localization. Such variants may alter ion-binding residues, pore architecture, conformational transitions, or regulatory interfaces, thereby reducing transmembrane ion flux despite apparently preserved expression [41,42]. Several CLCNKB missense variants fit this model, because mature channels can still be detected at the basolateral membrane while chloride conductance is markedly reduced [39]. Even here, however, genotype–phenotype interpretation should remain cautious. Functional deficits measured in vitro do not always translate linearly into clinical severity, and residual activity, modifier genes, developmental stage, and treatment history may all influence the phenotype. Thus, direct functional impairment is a useful mechanistic label, but it does not by itself fully explain the observed clinical heterogeneity of BS.

Taken together, current mechanistic studies strongly support the concept that BS-associated variants disrupt renal salt handling through multiple molecular routes, including protein misfolding, ER retention, ERAD, abnormal trafficking, splice defects, and impaired channel or transporter function. However, the evidence base remains fragmented. Many published studies focus on a small number of variants, use nonrenal model systems, and evaluate biochemical endpoints without long-term clinical correlation. Future work will need to integrate variant-specific functional assays, patient-derived cellular models, and longitudinal phenotyping to determine which mechanistic categories are most predictive of disease severity and therapeutic responsiveness.

## 3. Clinical Phenotypes and Genotype–Phenotype Correlations

### 3.1. Spectrum of Clinical Manifestations

Bartter syndrome (BS) encompasses a complex group of inherited renal tubular disorders fundamentally characterized by hypokalemia and metabolic alkalosis, but the relationship between genotype and phenotype is not strictly deterministic. Although certain genes are associated with recognizable clinical patterns, substantial overlap exists across subtypes, and the same gene may give rise to markedly different presentations depending on variant type, residual protein function, developmental stage, and modifying genetic or environmental influences. Recognizing this variability is critical for accurate clinical classification and individualized management.

#### 3.1.1. Bartter Syndrome Type I (BS I)

Caused by mutations in the SLC12A1 gene encoding the NKCC2 cotransporter, BS I often presents with a severe antenatal or neonatal phenotype, including polyhydramnios, prematurity, dehydration, growth impairment, and early-onset hypokalemic alkalosis. However, the clinical course is not completely uniform. Disease severity may vary according to the nature of the underlying variants and the degree of residual transporter activity, and not all patients exhibit the full classic phenotype. Accordingly, SLC12A1-associated disease should be regarded as typically severe rather than invariable in presentation, as shown in Table 2.

#### 3.1.2. Bartter Syndrome Type II (BS II)

BS II results from mutations in the KCNJ1 gene, which encodes the ROMK potassium channel. The classic description includes transient neonatal hyperkalemia followed by persistent hypokalemic metabolic alkalosis [43,44]. Nevertheless, this pattern should not be interpreted as obligatory. Atypical and late-onset phenotypes have been reported, and some patients present predominantly with nephrocalcinosis or an incomplete biochemical picture rather than the full biphasic pattern. These observations indicate that KCNJ1-associated disease is clinically broader than the traditional textbook description and that isolated hallmark features should not be overinterpreted in the absence of molecular confirmation [45,46].

#### 3.1.3. Bartter Syndrome Type III (BS III)

Associated with mutations in the CLCNKB gene encoding the ClC-Kb chloride channel, BS III exhibits one of the broadest phenotypic spectra within the BS group. Clinical manifestations may range from severe neonatal disease resembling BS I or II to milder or late-onset presentations that only become apparent in adolescence or adulthood [31,39,47].

Importantly, some adult-onset or attenuated cases of CLCNKB-related disease clinically resemble Gitelman syndrome, highlighting the limited specificity of phenotype-based classification. Although different CLCNKB variants may exert distinct effects on channel expression, trafficking, or function, current evidence does not always permit a precise prediction of clinical severity from genotype alone. Molecular testing is therefore essential not only for classification but also because the observed phenotype may underestimate the underlying genetic complexity [16].

#### 3.1.4. BS Type IV, MAGED2-Related Transient Antenatal Disease, and CaSR-Associated Bartter-like Phenotypes

BS type IV remains distinguished by the presence of severe renal salt wasting together with sensorineural deafness, reflecting the dual expression of barttin in both the kidney and inner ear. In addition, MAGED2 mutations define a distinct X-linked transient antenatal Bartter syndrome characterized by severe polyhydramnios, prematurity, and marked fetal or neonatal salt wasting, followed by partial or complete postnatal resolution in many survivors [7,8]. By contrast, activating (gain-of-function) mutations in the CASR gene may produce hypokalemic metabolic alkalosis together with hypocalcemia and hypercalciuria, but these cases overlap substantially with CaSR-related disorders of calcium homeostasis and are therefore better regarded as Bartter-like phenotypes rather than a canonical BS subtype [48,49,50].

Understanding these characteristic phenotypes remains clinically useful, but they should be interpreted as probabilistic patterns rather than rigid diagnostic rules. In practice, substantial overlap, age-dependent evolution, and incomplete phenotypic expression may blur the distinction between subtypes. A comprehensive view of this spectrum is therefore important not only for early recognition but also for avoiding overconfident phenotype-based classification in the absence of molecular confirmation [4,18,31].

### 3.2. Determinants of Phenotypic Severity

The severity of clinical manifestations in Bartter syndrome (BS) is not uniform and is influenced by the specific nature of the underlying variants, residual protein function, allelic combination, modifier genes, developmental timing, and environmental factors [51,52]. Although genotype–phenotype analysis is clinically informative, its predictive value remains incomplete. In many cases, currently available data support broad trends rather than precise prognostic rules, and this limitation should be acknowledged when interpreting mutation-specific severity.

#### 3.2.1. Impact of Truncating Mutations

The type of variant often influences disease severity. Truncating mutations, including nonsense variants, frameshift mutations, and whole-gene deletions, are generally more likely to cause severe loss of function than missense variants. However, this trend is not absolute. Some missense variants may be functionally severe, whereas some truncating alleles may be partially rescued by transcript or protein-level mechanisms. For example, truncating CLCNKB variants are often associated with earlier onset and more pronounced clinical manifestations, but considerable inter-individual variability remains even within this group [29,36].

#### 3.2.2. Allelic Heterogeneity and Compound Heterozygosity

Phenotypic expression is further modulated by allelic heterogeneity and compound heterozygosity. In many patients, the combination of a severe allele with a milder allele may produce an intermediate phenotype, consistent with partial residual function. However, even this model has limitations, because similar allelic combinations may still yield divergent clinical courses. These observations indicate that comprehensive genotyping is essential for diagnosis, but not always sufficient for precise prognostic prediction without functional and longitudinal clinical correlation.

#### 3.2.3. Modifier Genes and Environmental Factors

Beyond the primary causative mutations, the patient’s overall genetic background and various environmental factors contribute significantly to the phenotypic variability of BS. Modifier genes can substantially influence the baseline expression levels and functional capacity of renal transporters and ion channels, thereby intrinsically altering the severity of the disease [19,53,54]. Concurrently, environmental factors play a highly active role in modifying the clinical presentation. Variations in the dietary intake of sodium and potassium, as well as exposure to specific medications, can directly impact the patient’s fragile electrolyte balance. These external modifiers help to explain the clinical phenomenon where patients with identical SLC12A1, KCNJ1, or CLCNKB genotypes may ultimately exhibit highly divergent clinical courses over time. Recognizing the combined impact of primary mutations, genetic modifiers, and environmental influences is essential for optimizing therapeutic strategies and providing truly personalized medicine for patients with Bartter syndrome [5,35,55]. To synthesize these concepts, Table 3 summarizes representative relationships among mutation type, molecular mechanism, phenotypic expression, and potential therapeutic relevance across major BS disease entities.

## 4. Advances in Molecular Diagnostics

### 4.1. Genomic Sequencing Technologies

The molecular genetic diagnosis of Bartter syndrome (BS) has evolved significantly through the integration of both traditional and advanced high-throughput sequencing technologies, each providing distinct clinical utilities and addressing specific diagnostic limitations [35,56,57].

#### 4.1.1. Traditional Sanger Sequencing

Traditional Sanger sequencing remains a highly accurate and reliable diagnostic tool, particularly in clinical scenarios where a clear family history exists or specific clinical features strongly implicate a single candidate gene [58,59,60]. However, its inherently low throughput and labor-intensive methodology substantially limit its utility as a primary screening method for a genetically heterogeneous condition like BS, where multiple genes and complex structural variants are frequently implicated. Despite these limitations, Sanger sequencing remains an indispensable tool for confirming suspected variants identified by broader screening methods; for instance, it has been effectively utilized to validate novel complex heterozygous mutations within the CLCNKB gene in cases of adult-onset type III BS [61], as shown in Table 4.

#### 4.1.2. Targeted Gene Panel Sequencing

Given the profound genetic heterogeneity and the extensive phenotypic overlap among BS subtypes and related tubulopathies, targeted gene panel sequencing has firmly established itself as the frontline diagnostic tool. This high-throughput approach allows for the simultaneous and rapid analysis of dozens of genes associated with BS, Gitelman syndrome, and other inherited renal tubular disorders. By efficiently capturing both single nucleotide variants and small insertions or deletions across multiple genes in a single, cost-effective assay, targeted panels streamline the diagnostic workflow and directly address the challenge of phenotypic ambiguity. Notably, clinical studies have demonstrated that targeted panels can achieve a diagnostic yield as high as 44% in patients clinically diagnosed with classic BS who previously tested negative for CLCNKB mutations. This broad screening capability is vital for uncovering alternative genetic etiologies and correcting clinical misdiagnoses, such as cases initially mistaken for Dent disease or Geller syndrome [29].

#### 4.1.3. Whole-Exome Sequencing (WES) and Whole-Genome Sequencing (WGS)

**Figure 2:**
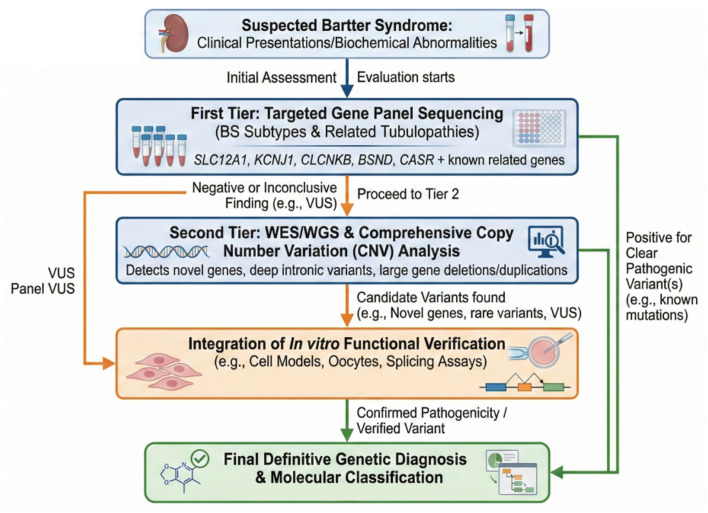
Comprehensive Diagnostic Algorithm for Bartter Syndrome as a professional scientific flowchart.

In complex clinical scenarios where targeted panel sequencing fails to yield a conclusive diagnosis, or when patients present with highly atypical phenotypes, whole-exome sequencing (WES) and whole-genome sequencing (WGS) provide comprehensive, genome-wide coverage. While WES focuses efficiently on the protein-coding regions (exons) to capture the vast majority of known disease-causing variants, WGS extends this analytical capacity deep into non-coding regions. This is particularly critical for identifying deep intronic sequences that may drastically affect gene regulation or pre-mRNA splicing. A prime example highlighting the power of WGS in BS diagnostics is the recent identification of a novel deep intronic mutation (c.629-527 T > C) in the SLC12A1 gene [62]. Subsequent minigene assays confirmed that this variant severely disrupted normal splicing, contributing directly to BS type I pathogenesis, a finding that would have been entirely missed by standard targeted panels or WES. Furthermore, both WES and WGS significantly enhance the detection of complex structural variants, such as the large gene deletions or duplications that represent major contributors to BS genetics. Ultimately, the strategic integration of traditional Sanger sequencing for validation, targeted panels for frontline screening, and comprehensive WES/WGS for unresolved cases forms a highly effective tiered diagnostic strategy. This structured approach brilliantly balances diagnostic accuracy, operational efficiency, and genomic breadth, thereby enabling precise molecular classification to guide personalized therapeutic decisions [63], as shown in Figure 2.

### 4.2. Diagnostic Challenges

Despite the rapid evolution of high-throughput sequencing technologies, diagnosing Bartter syndrome (BS) remains fraught with significant clinical and molecular challenges. These diagnostic hurdles primarily stem from the profound phenotypic overlap among renal tubulopathies, the complex interpretation of novel genetic variants, and the technical difficulties inherent in detecting structural genomic anomalies [26], as shown in Table 5.

#### 4.2.1. Phenotypic Overlap with Other Tubulopathies

One of the most formidable clinical challenges is the extensive phenotypic overlap that exists not only among the various BS subtypes but also with other inherited salt-losing tubulopathies, most notably Gitelman syndrome and renal tubular acidosis. Because the core clinical and biochemical features, such as hypokalemia, metabolic alkalosis, and elevated renin-aldosterone levels, are commonly shared across these distinct disorders, achieving an accurate diagnosis based solely on biochemical parameters is exceptionally difficult. A prime example is BS type III, caused by mutations in the CLCNKB gene; adult-onset or milder cases of BS type III frequently present with symptoms that are clinically indistinguishable from Gitelman syndrome. This striking phenotypic ambiguity underscores the absolute necessity of integrating comprehensive genetic testing to achieve an accurate molecular classification, which is ultimately critical for determining patient prognosis and guiding appropriate clinical management [31,47].

#### 4.2.2. Interpretation of Variants of Uncertain Significance (VUS)

As next-generation sequencing becomes more widely implemented, the subsequent discovery of new DNA sequence changes has introduced another major diagnostic bottleneck: the interpretation of novel variants and variants of uncertain significance (VUS). Determining the true pathogenicity of these newly identified variants requires a highly rigorous and comprehensive evaluation framework. This process must integrate family segregation studies, population frequency databases, and in silico predictive algorithms; however, these predictive models are often insufficient on their own. Definitive classification frequently relies on robust functional validation techniques, such as patch-clamp electrophysiology, immunofluorescence localization, and Western blotting, to precisely assess the functional impact of the variant on the protein’s cellular activity. For instance, highly specialized minigene splicing assays have been instrumental in elucidating the pathogenic effects of novel splice-site mutations identified within the SLC12A1 and CLCNKB genes, definitively confirming their role in aberrant mRNA processing and BS pathogenesis [40]. Moving beyond predictive models to functional evidence is essential for resolving VUS and ensuring diagnostic accuracy.

#### 4.2.3. Detection of Copy Number Variations (CNVs)

The detection of copy number variations (CNVs), which include whole-gene deletions or duplications, presents a significant technical challenge in the molecular diagnosis of BS. CNVs are substantial contributors to the genetic landscape of BS, but they are frequently overlooked if sequencing data pipelines are strictly optimized to analyze only single nucleotide variants.

For example, large whole-gene deletions of the CLCNKB gene have been identified as highly common pathogenic events driving type III BS [30,37]. These critical structural variants often evade detection by standard sequencing unless specific complementary techniques, such as quantitative PCR or sophisticated read-depth analysis of sequencing data, are deliberately employed. Therefore, explicitly incorporating robust CNV analysis into routine genetic testing workflows is paramount to significantly enhance the overall diagnostic yield and prevent detrimental false-negative results, particularly in clinical cases where there is a high suspicion of BS despite negative findings for point mutations. To effectively overcome these multifaceted diagnostic challenges, clinicians and geneticists must adopt a holistic, multidisciplinary approach. This strategy requires integrating detailed clinical evaluations with advanced genomic sequencing (including WES or WGS with integrated CNV analysis) and rigorous, function-based variant interpretation to successfully navigate the complexities of inherited renal tubular disorders.

## 5. Current Management and Emerging Precision Therapies

### 5.1. Limitations of Conventional Treatments

The current standard of care for Bartter syndrome (BS) remains predominantly palliative, focusing primarily on symptomatic management rather than addressing the root molecular etiology. The cornerstone of this conventional approach involves rigorous electrolyte supplementation, specifically targeting potassium, sodium, and magnesium, to counteract the chronic renal salt wasting and profound hypokalemia characteristic of the disease [64]. Concurrently, nonsteroidal anti-inflammatory drugs (NSAIDs), predominantly indomethacin, are widely prescribed to inhibit cyclooxygenase enzymes. This pharmacological inhibition suppresses the excessive renal synthesis of prostaglandin E2 (PGE2), thereby mitigating polyuria, curtailing further electrolyte depletion, and supporting patient growth and metabolic stability. This dual therapeutic strategy is particularly utilized in the management of antenatal and classic BS phenotypes, specifically types I, II, and III.

Despite providing essential symptomatic relief, these conventional therapies are fundamentally limited by their inability to correct the underlying genetic defects or restore the functional integrity of the defective ion transporters within the thick ascending limb. Consequently, patients are burdened with a requirement for lifelong, continuous pharmacological management. Because the core pathophysiological process persists unabated, patients remain highly susceptible to severe, long-term clinical complications, including the development of progressive nephrocalcinosis, chronic kidney disease (CKD), and sustained growth retardation. Furthermore, the extreme genetic heterogeneity and phenotypic variability inherent to BS severely complicate the establishment of standardized treatment protocols, as individual therapeutic responses can fluctuate widely depending on the patient’s specific mutational profile.

The chronic administration of these conventional therapies also introduces significant clinical risks and tolerability issues. Long-term NSAID therapy is fraught with substantial adverse effects, most notably a high risk of nephrotoxicity, severe gastrointestinal complications, and potential cardiovascular toxicity. These inherent risks necessitate the vigilant and continuous monitoring of renal function and overall patient health. Moreover, the therapeutic efficacy of NSAIDs is not uniform across all BS classifications; for instance, their clinical benefit is considerably less pronounced in BS type IV or in patients exhibiting highly atypical clinical presentations [65].

Similarly, the aggressive electrolyte supplementation required to manage BS is frequently insufficient to fully normalize serum electrolyte concentrations due to the relentless nature of the ongoing renal losses. The remarkably high oral doses necessitated by this continuous depletion are notoriously difficult for patients to maintain and frequently precipitate severe gastrointestinal intolerance, further compromising patient compliance and overall quality of life. Ultimately, the profound limitations, adverse side effects, and strictly palliative nature of these current strategies emphatically highlight the urgent clinical need for novel, disease-modifying therapeutic approaches that target the specific molecular mechanisms of the syndrome.

### 5.2. Targeted and Genotype-Specific Interventions

Recent advances in molecular genetics and cellular biology have broadened interest in targeted therapies designed to correct or mitigate the molecular defects underlying Bartter syndrome (BS) [5]. However, most of these approaches remain preclinical, and their translational status varies substantially across therapeutic categories. Accordingly, they should be discussed as emerging experimental strategies rather than as clinically validated precision treatments.

#### 5.2.1. Molecular Chaperone Therapy for Protein Misfolding: Experimental Promise but No Clinical Validation Yet

A significant proportion of BS cases, particularly those caused by missense mutations in the SLC12A1 (encoding NKCC2) and KCNJ1 (encoding ROMK) genes, result in synthesized transport proteins that are severely misfolded [5]. These defective proteins are subsequently retained within the endoplasmic reticulum (ER) and prematurely destroyed via the ER-associated degradation (ERAD) pathway, leading to a profound loss of function at the apical membrane.

To combat this specific pathogenic mechanism, molecular chaperone therapy has emerged as a highly promising targeted intervention. Chemical chaperones, such as 4-phenylbutyric acid (4-PBA), act as pharmacological agents that assist in stabilizing and correctly folding these mutant proteins. In vitro functional studies utilizing HEK293 cell models have explicitly demonstrated that treatment with 4-PBA successfully improves the folding efficiency of specific, trafficking-defective NKCC2 mutants, such as the SLC12A1 p.L479V variant [66]. By rescuing these proteins from ER retention, 4-PBA significantly increases their proper trafficking, enhances mature protein expression at the plasma membrane, and partially restores their essential ion transport capabilities. Immunofluorescence assays have further confirmed that 4-PBA effectively reduces the cytoplasmic retention of these mutant cotransporters, establishing a powerful proof-of-concept for genotype-specific pharmacological rescue [67]. While currently limited to cellular models, the continued development of more potent and highly specific chaperones holds immense potential for clinical translation.

#### 5.2.2. RNA-Targeted Therapies and Antisense Oligonucleotides (ASOs)

Beyond protein misfolding, complex mRNA splicing abnormalities, often triggered by splice-site mutations identified in genes like CLCNKB and SLC12A1, represent another critical molecular target in BS pathogenesis. For patients harboring these specific defects, RNA-targeted therapies, particularly antisense oligonucleotides (ASOs), offer a cutting-edge precision medicine approach.

ASOs are synthetically engineered nucleic acid sequences designed to bind to specific target pre-mRNAs. By doing so, they can therapeutically modulate the splicing machinery to correct aberrant splicing events, such as deleterious exon skipping or pathogenic intron retention. This targeted modulation effectively restores the production of normal mRNA transcripts and ensures the subsequent translation of fully functional transport proteins. Representing a highly personalized treatment modality, ASO therapy directly addresses the unique splicing defects of individual patients and leverages therapeutic strategies that have already demonstrated remarkable clinical success in other severe genetic disorders.

#### 5.2.3. Gene Therapy and CRISPR/Cas9 Gene Editing: Conceptually Curative but Still Preclinical

In principle, gene therapy and advanced genome-editing technologies represent the most direct route toward causal correction of the underlying molecular defect. Techniques such as CRISPR/Cas9 are theoretically capable of correcting pathogenic DNA variants or enabling the delivery of functional gene copies to restore transporter activity [68,69,70]. However, in the context of Bartter syndrome, this concept remains largely hypothetical and preclinical rather than an imminent therapeutic reality.

Major physiological and technical barriers continue to limit the translational feasibility of these approaches in BS. The highly compartmentalized structure of the kidney makes safe and efficient delivery to the relevant renal tubular cells particularly difficult. In addition, off-target editing, incomplete editing efficiency, durability of correction, vector-related toxicity, and potential immune responses remain unresolved concerns. At present, there is no established gene-editing therapy for Bartter syndrome in clinical use, and the field remains at an exploratory preclinical stage. Therefore, CRISPR/Cas9 should be discussed as a promising research platform rather than as a near-term curative treatment.

## 6. Future Perspectives and Conclusion

The intricate relationship between genotype and phenotype in Bartter syndrome (BS) remains a highly complex and critical area of investigation. To truly unravel this complexity and move beyond the current diagnostic limitations, establishing large-scale international patient cohorts that meticulously integrate comprehensive clinical phenotyping with detailed genotypic data is absolutely essential. The future application of machine learning and advanced bioinformatics to these massive, integrated datasets will significantly enhance our predictive accuracy for disease progression, the risk of severe complications, and individual therapeutic responsiveness. Furthermore, extensively mapping the influence of genetic modifiers and environmental factors through these large-scale cohorts will enable highly personalized prognostic counseling and tailored lifestyle recommendations.

In the realm of direct clinical care, the inherent complexity and phenotypic overlap of BS necessitate the rigorous optimization of management pathways. A fundamental cornerstone of this optimized care is the establishment of comprehensive multidisciplinary teams (MDTs). Comprising specialized nephrologists, genetic counselors, endocrinologists, and otolaryngologists (particularly essential for managing the sensorineural deafness in BS type IV), these MDTs are critical for holistic and effective patient management.

An integrated MDT can seamlessly coordinate the entire continuum of care, from initial genetic evaluation and complex diagnosis to personalized treatment planning and vigilant long-term monitoring for complications such as chronic kidney disease and nephrocalcinosis.

Translational medicine serves as the vital bridge connecting foundational genetic discoveries with the realization of precision therapeutics. The integration of high-throughput drug screening platforms, utilizing advanced models such as patient-derived renal cells, organoids, and engineered yeast platforms, offers highly promising avenues to rapidly identify small molecules capable of stabilizing mutant proteins or augmenting residual transport function. However, translating these scientific advances into clinical practice requires robust preclinical validation and exceptionally well-designed clinical trials to stringently assess the safety and efficacy of emerging treatments, including chemical chaperones, RNA-targeted antisense oligonucleotides (ASOs), and future gene-editing technologies.

In conclusion, molecular genetic research on Bartter syndrome has made remarkable strides, successfully elucidating the core BS genes SLC12A1, KCNJ1, CLCNKB, BSND, and MAGED2 and their corresponding functional defects, while clarifying that CaSR-related disease is more appropriately considered a related Bartter-like phenotype than a stable canonical BS subtype. Nevertheless, completely bridging the gap between genetic diagnosis and clinical manifestation requires an integrative approach that intimately combines high-throughput sequencing technologies with rigorous in vitro functional studies. The identification of specific molecular defects, such as endoplasmic reticulum-associated degradation (ERAD) and aberrant mRNA splicing, has illuminated highly promising therapeutic targets.

The future of Bartter syndrome management firmly relies on a paradigm shift toward precision medicine, fundamentally tailored to the specific molecular pathology of individual patients. Achieving these ambitious goals, transitioning from merely palliative symptomatic relief to definitive mechanism-based correction, necessitates sustained international collaboration, the seamless integration of multidisciplinary clinical expertise, and an unwavering commitment to translational medical research.

## 7. Conclusions

Molecular genetic research on Bartter syndrome has made remarkable strides, elucidating the principal BS genes SLC12A1, KCNJ1, CLCNKB, BSND, and MAGED2 and their corresponding protein defects, thereby refining the molecular framework of disease classification while separating CaSR-associated Bartter-like disease from the core canonical BS spectrum. This progress has significantly deepened our understanding of the underlying pathophysiology and provided an essential framework for correlating genotypes with clinical phenotypes. However, the intricate relationship between genetic mutations and clinical manifestations remains complex and multifaceted, reflecting the profound heterogeneity of the syndrome. Addressing these diagnostic challenges and refining disease classification beyond traditional clinical criteria requires an integrative approach that seamlessly balances high-throughput sequencing technologies with rigorous functional studies.

The mechanisms by which these genetic mutations lead to protein dysfunction are diverse, encompassing critical defects in protein expression, impaired membrane localization, and direct functional impairments. Notably, aberrant protein folding, endoplasmic reticulum-associated degradation (ERAD), and splicing abnormalities have emerged as critical pathogenic pathways. These mechanistic insights not only enhance our fundamental understanding of the disease but also highlight highly promising therapeutic targets. While current treatments remain predominantly symptomatic, focusing primarily on managing electrolyte imbalances and associated complications, they inherently fail to address the underlying molecular defects driving the disease.

The precise identification of specific molecular defects opens innovative avenues for the development of targeted interventions aimed at correcting or compensating for specific protein abnormalities. For instance, molecular chaperones that assist in protein folding, agents that modulate aberrant splicing, and future gene-based strategies represent important experimental directions for mechanism-based therapy. Consequently, the future of Bartter syndrome management may increasingly move toward precision medicine tailored to the molecular pathology of individual patients. However, the transition from concept to clinical implementation will require substantial additional functional, translational, and trial-level evidence. Such mechanism-based strategies promise not only to alleviate clinical symptoms but to fundamentally modify disease progression, thereby drastically improving long-term prognosis and quality of life for patients.

Achieving these transformative goals necessitates sustained international collaboration, which facilitates the crucial sharing of genetic data, clinical experiences, and research resources. Furthermore, in-depth functional studies remain indispensable to rigorously validate the pathogenicity of novel mutations and elucidate their biological consequences. Overcoming the translational gap requires the deep integration of multidisciplinary expertise, spanning molecular genetics, cell biology, clinical nephrology, and pharmacology, coupled with well-designed clinical trials to stringently assess safety and efficacy. Ultimately, continued collaborative efforts and dedicated translational research are imperative to realize the full potential of these scientific advances and significantly improve outcomes for patients afflicted with this challenging disorder.

## Figures and Tables

**Table 1:** Updated Genetic Framework and Associated Transport Defects in Bartter Syndrome and Related Bartter-like Phenotypes.

Subtype	Gene	Locus	Encoded Protein	Membrane Localization	Primary Function
Type I BS	SLC12A1	Not specified	Na^+^-K^+^-2Cl^−^ cotransporter (NKCC2)	Apical membrane of TAL cells	Mediates the reabsorption of sodium, potassium, and chloride ions from the tubular lumen; essential for corticomedullary osmotic gradient generation.
Type II BS	KCNJ1	Not specified	Renal outer medullary potassium channel (ROMK)	Apical membrane of TAL cells and collecting duct principal cells	Facilitates potassium recycling into the tubular lumen to sustain NKCC2 activity and maintain potassium secretion.
Type III BS	CLCNKB	Not specified	Voltage-gated chloride channel (ClC-Kb)	Basolateral membrane of TAL cells and distal convoluted tubule	Mediates chloride exit from tubular cells into the interstitium; critical for maintaining electroneutrality during sodium chloride reabsorption.
Type IV BS	BSND	Not specified	Barttin (accessory β-subunit)	Renal tissues and inner ear	Essential for the proper membrane localization and physiological function of ClC-Ka and ClC-Kb channels.
MAGED2-related transient antenatal BS	MAGED2	Xp11.21	Melanoma-associated antigen D2 (MAGE-D2)	Intracellular regulatory pathway linked to TAL and distal tubular transport	Supports the trafficking and functional expression of NKCC2/NCC during fetal development; defects cause severe antenatal salt wasting with often transient postnatal resolution.
CaSR-associated Bartter-like phenotype (historically termed “type V BS”)	CASR	3q13.3-q21	Calcium-sensing receptor (CaSR)	Renal and parathyroid tissues	Activating variants alter calcium sensing and may produce a Bartter-like tubulopathy with hypocalcemia and hypercalciuria; increasingly viewed within the broader spectrum of calcium-homeostasis disorders.

**Table 2:** Typical Genotype–Phenotype Patterns, Variability, and Distinctive Clinical Features in Bartter Syndrome.

BS Subtype	Typical Age of Onset	Common Clinical Presentations	Characteristic but Variable Features	Usual Phenotypic Severity
Type I BS (*SLC12A1*)	Antenatal to neonatal	Polyhydramnios, fetal polyuria, premature delivery, profound dehydration, and early-onset hypokalemic alkalosis.	Severe antenatal manifestations.	Consistently severe.
Type II BS (*KCNJ1*)	Neonatal, with rare late-onset presentations	Transient neonatal hyperkalemia followed by persistent hypokalemia, and hypokalemic metabolic alkalosis.	High incidence of nephrocalcinosis due to disturbed calcium handling.	Generally severe, though atypical phenotypes dominated by nephrocalcinosis exist.
Type III BS (*CLCNKB*)	Highly variable, ranging from severe neonatal onset to adulthood	Hypokalemia, metabolic alkalosis, and growth retardation.	Broadest phenotypic spectrum among subtypes; adult-onset cases often clinically resemble Gitelman syndrome; nephrocalcinosis is less frequent than in types I and II.	Ranging from mild to severe.
Type IV BS (*BSND*)	Antenatal/Neonatal	Classical renal salt wasting phenotype.	Concomitant sensorineural deafness due to barttin expression in the inner ear.	Severe.
Type V BS (*CASR*)	Variable	Biochemical abnormalities closely resembling classic Bartter syndrome.	Presence of hypocalcemia; uniquely distinguished by an autosomal dominant inheritance pattern.	Variable.

**Table 3:** Representative Relationships among Mutation Type, Molecular Mechanism, Phenotypic Expression, and Therapeutic Implications in Bartter Syndrome.

Gene/Disease Entity	Representative Mutation Type	Dominant Molecular Consequence	Typical Phenotypic Pattern	Usual Severity Trend	Potential Therapeutic Relevance
SLC12A1 (BS I)	Missense variants causing folding defects; splice-site variants; truncating variants	ER retention, ER-associated degradation (ERAD), impaired apical trafficking, or loss of NKCC2 transport activity	Antenatal/neonatal salt wasting, polyhydramnios, prematurity, severe dehydration, early hypokalemic alkalosis	Usually severe, but variable according to residual function	Selected trafficking-defective variants may be candidates for experimental chaperone rescue; splice-altering variants may inform future RNA-based strategies
KCNJ1 (BS II)	Missense variants affecting folding or gating; truncating variants	Misfolding with intracellular degradation, reduced membrane expression, or defective ROMK channel activity	Transient neonatal hyperkalemia followed by hypokalemic alkalosis; nephrocalcinosis is common; atypical cases occur	Often severe in infancy, but phenotype is variable	Mechanism-specific rescue strategies remain experimental; functional classification may help stratify variants
CLCNKB (BS III)	Missense variants, splice variants, whole-gene deletions, complex rearrangements	Reduced membrane expression, trafficking defects, splicing abnormalities, or impaired chloride conductance	Broad clinical spectrum from neonatal disease to adult presentations mimicking Gitelman syndrome	Highly variable	Variant type may inform expected residual function, but prognostic precision remains limited
BSND (BS IV)	Loss-of-function variants	Defective barttin-dependent membrane localization and function of ClC-K channels	Severe renal salt wasting with sensorineural deafness	Usually severe	Primarily supportive management; useful model for channel assembly-based therapeutic research
MAGED2-related transient antenatal BS	Loss-of-function variants	Impaired regulatory support for NKCC2/NCC trafficking and expression during fetal life	Severe fetal polyhydramnios, prematurity, marked antenatal salt wasting with frequent postnatal improvement	Very severe prenatally, often transient after birth	Highlights developmental and context-dependent mechanisms rather than classic structural transporter defects
CaSR-associated Bartter-like phenotype	Activating missense variants	Enhanced CaSR signaling with altered calcium and salt handling	Hypokalemic metabolic alkalosis with hypocalcemia and hypercalciuria in selected patients	Variable	Therapeutic interpretation should consider overlap with calcium-homeostasis disorders rather than canonical BS alone

**Table 4:** Comparison of Molecular Diagnostic Technologies for Renal Tubulopathies.

Sequencing Technology	Primary Target	Advantages	Limitations in BS Diagnosis
Sanger Sequencing	Specific candidate genes or known familial variants.	High accuracy and reliability; excellent for confirming suspected variants or validating complex heterozygous mutations.	Low throughput and labor-intensive; limited utility as an initial screening tool for highly heterogeneous conditions like BS.
Targeted Gene Panel Sequencing	Dozens of known genes associated with BS and related renal tubular disorders (e.g., Gitelman syndrome).	High diagnostic efficiency; effectively addresses phenotypic overlap; simultaneously detects single nucleotide variants and small insertions/deletions across multiple genes.	Cannot identify variants in novel genes not included in the specific panel; may miss deep intronic mutations.
Whole-Exome Sequencing (WES)	Protein-coding regions (exons) across the entire genome.	Captures the vast majority of disease-causing variants; facilitates the detection of complex structural variants and copy number variations (CNVs) with appropriate analysis.	Fails to capture non-coding regions, potentially missing deep intronic sequences that affect gene regulation or splicing.
Whole-Genome Sequencing (WGS)	Comprehensive genomic coverage, including both coding and non-coding regions.	Capable of detecting deep intronic sequences that disrupt normal splicing (e.g., novel SLC12A1 variants); superior for identifying complex structural variants.	Generates vast amounts of data, making the interpretation of variants of uncertain significance (VUS) highly complex and resource-intensive.

**Table 5:** In Vitro Functional Verification Models for Bartter Syndrome Mutations.

Experimental Model	Key Applications	Advantages	Limitations
Mammalian Cell Lines (e.g., HEK293, MDCK)	Analyzing protein expression levels, glycosylation maturation status, and subcellular localization using Western blotting and immunofluorescence.	Ease of culture and transient or stable transfection; provides direct visual and biochemical evidence of disrupted protein biosynthesis, ER retention, or membrane trafficking.	Generally lower throughput compared to yeast platforms; more challenging to assess precise ion channel gating properties and selectivity than in oocyte systems.
Xenopus laevis Oocyte Expression System	Precise electrophysiological measurements utilizing two-electrode voltage clamp techniques to assess ionic currents, channel conductance, and specific transporter activity.	Highly informative for quantifying the exact degree of functional loss; uniquely facilitates the detailed study of gating properties and ion selectivity that are difficult to capture in standard mammalian cell lines.	Requires highly specialized electrophysiology equipment and technical expertise; inherently lower throughput for screening multiple variants.
Yeast High-Throughput Screening Platforms	Rapid functional assessment of large variant libraries derived from genomic data; utilizing cell growth or survival under selective conditions as a proxy for protein biogenesis and function.	Exceptional scalability and cost-effectiveness for screening numerous mutations simultaneously; highly useful for dissecting fundamental protein folding and degradation pathways.	Provides proxy measurements rather than direct electrophysiological data; complements rather than replaces the detailed mechanistic analyses required in mammalian cells or oocytes.

## Data Availability

No new data were created during this study.

## Abbreviations

The following abbreviations are used in this manuscript:

BS	Bartter syndrome
ERAD	Endoplasmic Reticulum-Associated Degradation
ER	Endoplasmic Reticulum
